# Supplementary material for: Entire expressed peripheral blood transcriptome in pediatric severe malarial anemia
Source: Nat Commun. 2024 Jun 12;15:5037. doi: 10.1038/s41467-024-48259-4 (PMC11169501; doi:10.1038/s41467-024-48259-4)
Supplement: Supplementary file 1 — Supplementary Information [file 41467_2024_48259_MOESM1_ESM.pdf]

Supplementary Information

Entire Expressed Peripheral Blood Transcriptome in Pediatric Severe Malarial Anemia

Samuel B. Anyona[1,2]*, Qiuying Cheng[3], Sharley A. Wasena[2,4], Shamim W. Osata[2] , Yan Guo[5], Evans Raballah[2,6], Ivy Hurwitz[3], Clinton O. Onyango[2,4], Collins Ouma[2,4] , Philip D. Seidenberg[7], Benjamin H. McMahon[8], Christophe G. Lambert[9], Kristan A. Schneider[9,10] and Douglas J. Perkins[2,3]*

[1]Department of Medical Biochemistry, School of Medicine, Maseno University, Maseno 40105, Kenya;

[2]University of New Mexico-Kenya Global Health Programs, Kisumu and Siaya 40100, Kenya;

[3]Department of Internal Medicine, Center for Global Health, University of New Mexico, Albuquerque, New Mexico 87131-0001, USA;

[4]Department of Biomedical Sciences and Technology, School of Public Health and Community Development, Maseno University, Maseno 40105, Kenya;

[5]Department of Public Health Sciences, Biostatistics Division, University of Miami, Miami 33136, USA;

[6]Department of Medical Laboratory Sciences, School of Public Health, Biomedical Sciences and Technology, Masinde Muliro University of Science and Technology, Kakamega 50100, Kenya;

[7]Department of Emergency Medicine, School of Medicine, University of New Mexico, Albuquerque, New Mexico 87131-0001, USA;

[8]Theoretical Biology and Biophysics Group, Theoretical Division, Los Alamos National Laboratory, Los Alamos, New Mexico 87545, USA;

[9]Department of Internal Medicine, Division of Translational Informatics, University of New Mexico, Albuquerque, New Mexico 87131-0001, USA;

[10]Department Applied Computer and Bio-Sciences, University of Applied Sciences Mittweida, Mittweida 09648, Germany.

Corresponding Authors:

*Samuel B. Anyona, PhD, Department of Medical Biochemistry, School of Medicine, Maseno University, P. O. Box 333-40105, Maseno, Kenya. Phone: +254-721-543-976, Fax: +254-057-351-221, E-mail: sanyona@maseno.ac.ke

* Douglas Jay Perkins, PhD, Department of Internal Medicine, Center for Global Health, University of New Mexico, 915 Camino de Salud NE, IDTC 3120, Albuquerque, New Mexico 87131-0001, USA. Phone: +1 505-272-6867, Fax: +1 505-272-8441, E-mail: dperkins@salud.unm.edu

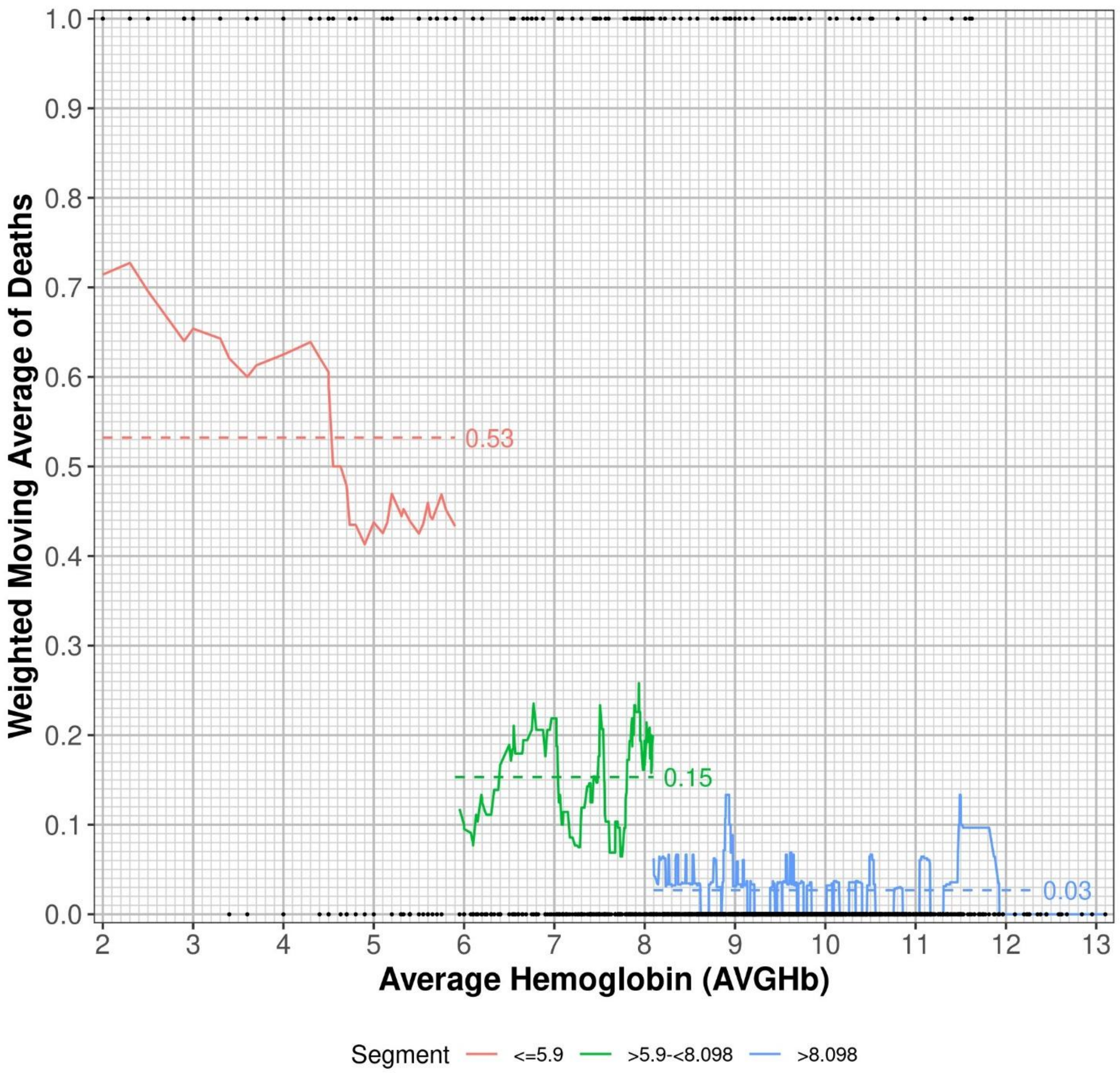

Fig. S1. Weighed moving average of mortality by average hemoglobin segments. Data were utilized from 1,644 children (3-48 mos.) who were followed for 36 mos. in which 19,231 Hb measurements were obtained. A dynamic programming approach was employed to test all possible splits from 2-way to 10-way exhaustively. The criterion for the optimal number of splits was the largest k for which all pairwise chi-square tests between resulting groups were significant (*p*<0.001). This approach yielded three distinct hemoglobin segments: (1) ≤5.9 g/dL (n=62, mortality fraction=0.53, shown in red), (2) 5.9-8.098 g/dL (n=209, mortality fraction=0.15, shown in green), (3) and >8.098 g/dL (n=1,373, mortality fraction=0.03, shown in blue). This data-driven approach that captured repeated hemoglobin concentrations at our study site in Siaya, western Kenya, formed the basis for selecting 6.0 g/dL as the criterion for life-threatening SMA.

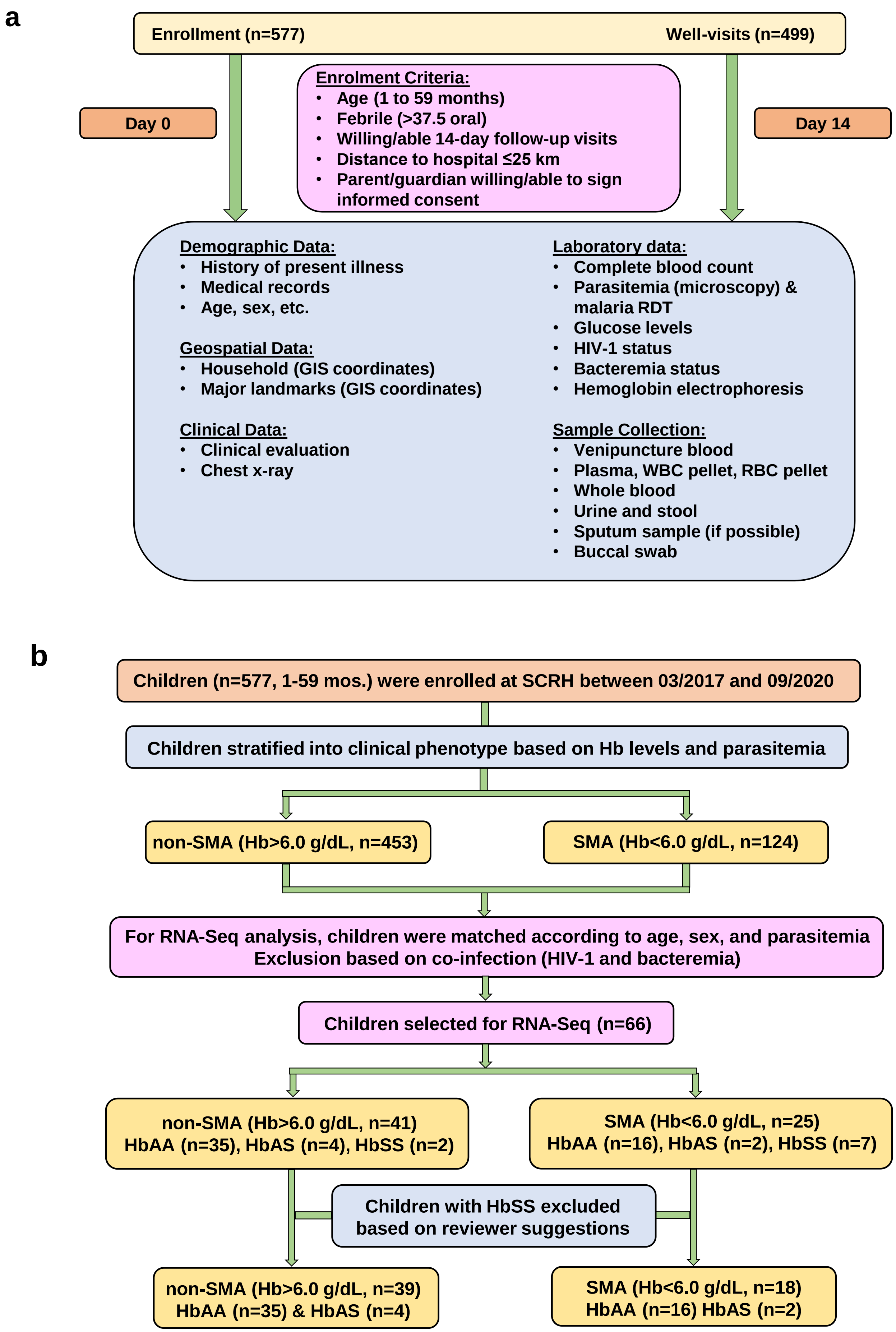
a
Enrollment (n=577)
Well-visits (n=499)
Day 0
Day 14
Enrolment Criteria:
• Age (1 to 59 months)
• Febrile (>37.5 oral)
• Willing/able 14-day follow-up visits
• Distance to hospital ≤25 km
• Parent/guardian willing/able to sign informed consent
Demographic Data:
• History of present illness
• Medical records
• Age, sex, etc.
Geospatial Data:
• Household (GIS coordinates)
• Major landmarks (GIS coordinates)
Clinical Data:
• Clinical evaluation
• Chest x-ray
Laboratory data:
• Complete blood count
• Parasitemia (microscopy) & malaria RDT
• Glucose levels
• HIV-1 status
• Bacteremia status
• Hemoglobin electrophoresis
Sample Collection:
• Venipuncture blood
• Plasma, WBC pellet, RBC pellet
• Whole blood
• Urine and stool
• Sputum sample (if possible)
• Buccal swab
b
Children (n=577, 1-59 mos.) were enrolled at SCRH between 03/2017 and 09/2020
Children stratified into clinical phenotype based on Hb levels and parasitemia
non-SMA (Hb>6.0 g/dL, n=453)
SMA (Hb<6.0 g/dL, n=124)
For RNA-Seq analysis, children were matched according to age, sex, and parasitemia
Exclusion based on co-infection (HIV-1 and bacteremia)
Children selected for RNA-Seq (n=66)
non-SMA (Hb>6.0 g/dL, n=41)
HbAA (n=35), HbAS (n=4), HbSS (n=2)
SMA (Hb<6.0 g/dL, n=25)
HbAA (n=16), HbAS (n=2), HbSS (n=7)
Children with HbSS excluded based on reviewer suggestions
non-SMA (Hb>6.0 g/dL, n=39)
HbAA (n=35) & HbAS (n=4)
SMA (Hb<6.0 g/dL, n=18)
HbAA (n=16) HbAS (n=2)

Fig. S2. Study design, sample collection, and sample selection strategy. The acute febrile cohort study was conducted at Siaya County Referral Hospital (SCRH), located in a holoendemic *P. falciparum* transmission region in western Kenya, where SMA is among the main causes of childhood morbidity and mortality. **(a)** The study enrolled 577 children (1-59 mos.) with 499 well visits (3/2017 to 9/2020). At enrollment (Day 0), demographic, geospatial, clinical, and laboratory data were collected. Prior to treatment with antimalarials or any other medications, venipuncture blood samples (3-4mL) were collected for laboratory measures. Parents/guardians were asked to return with their child to the hospital for a day 14 well visit. **(b)** There were 577 children enrolled between 03/2017 to 09/2020. Children were stratified into clinical phenotypes based on Hb levels and the presence of malaria parasitemia: non-SMA (Hb>6.0 g/dL, n=453) and SMA (Hb<6.0 g/dL, n=124). For the RNA-Seq analysis, children were matched according to age, sex, and parasitemia. Children with detected co-infections (i.e., HIV-1 and bacteremia) were excluded from sequencing. The study selected 66 children for RNA-Seq: non-SMA (Hb>6.0 g/dL, n=41 with sickle cell status as HbAA (n=35), HbAS (n=4), and HbSS (n=2), and SMA (Hb<6.0 g/dL, n=25 with sickle cell status as HbAA (n=16), HbAS (n=2), and HbSS (n=7). Based on suggestions from the journal referees, children with HbSS were then excluded from the final analyses. As such, the RNA-Seq data presented are for 39 children (3-48 mos.) with non-SMA [HbAA (n=35) and HbAS (n=4)] and 18 children with SMA [HbAA (n=16) HbAS (n=2)].

Table S1. Enrichment analysis of uniquely expressed DEGs.

#	Network	GO processes	Total nodes	Seed nodes	zScore	gScore	p-Value
	**Non-SMA**						
1	TFF3↔IL-6↔IL6RA↔ ADAM17↔gp130	T-helper 17 cell lineage commitment (50.0%; **2.841E-16**), T-helper 17 cell differentiation (50.0%; **8.324E-16**), Interleukin-6-mediated signaling pathway (50.0%; **1.669E-15**), T-helper cell lineage commitment (50.0%; **2.545E-15**), T-helper 17 type immune response (50.0%; **3.111E-15**)	14	3	167.21	167.21	**3.180E-12**
2	SHOX2↔Neuregulin 1↔ FGFR2↔Endothelin-1↔ECE2	Transmembrane receptor protein tyrosine kinase signaling pathway (62.1%; **7.814E-23**), Positive regulation of cell population proliferation (79.3%; **9.709E-23**), Enzyme-linked receptor protein signaling pathway (69.0%; **1.364E-22**), Positive regulation of phosphorylation (72.4%; **4.816E-22**), Fibroblast growth factor receptor signaling pathway (37.9%; **3.959E-21**)	29	2	77.44	77.44	**1.930E-07**
	**SMA**						
1	c-Myc↔C/EBPbeta↔STAT1↔ STAT5↔ERK1/2	Insulin-like growth factor receptor signaling pathway (16.0%; **1.290E-21**), Transmembrane receptor protein tyrosine kinase signaling pathway (28.4%; **4.394E-19**), Cellular response to peptide hormone stimulus (27.2%; **1.922E-18**), Enzyme-linked receptor protein signaling pathway (30.9%; **8.401E-17**), Cellular response to peptide (27.2%; **1.043E-16**)	90	58	425.02	425.02	**1.240E-200**
2	ACE1↔des-Arg9-bradykinin↔BDKRB1↔des-Arg10-kallidin↔BDKRB2	Positive regulation of cellular process (77.3%; **3.515E-15**), Transmembrane receptor protein tyrosine kinase signaling pathway (25.8%; **1.117E-13**), Cellular response to hormone stimulus (31.8%; **1.956E-13**), Response to chemical (69.7%; **2.395E-13**), Positive regulation of metabolic process (62.1%; **3.772E-13**)	79	33	258.26	258.26	**5.310E-103**

Enrichment analysis of uniquely expressed DEGs in non-SMA (n=602 genes) and SMA (n=493 genes) was performed using canonical pathway modeling in Metacore™ ($p<0.05$). Top-ranked Gene Ontology (GO) processes associated with each subnetwork are shown. Significant *p*-values are shown in bold.

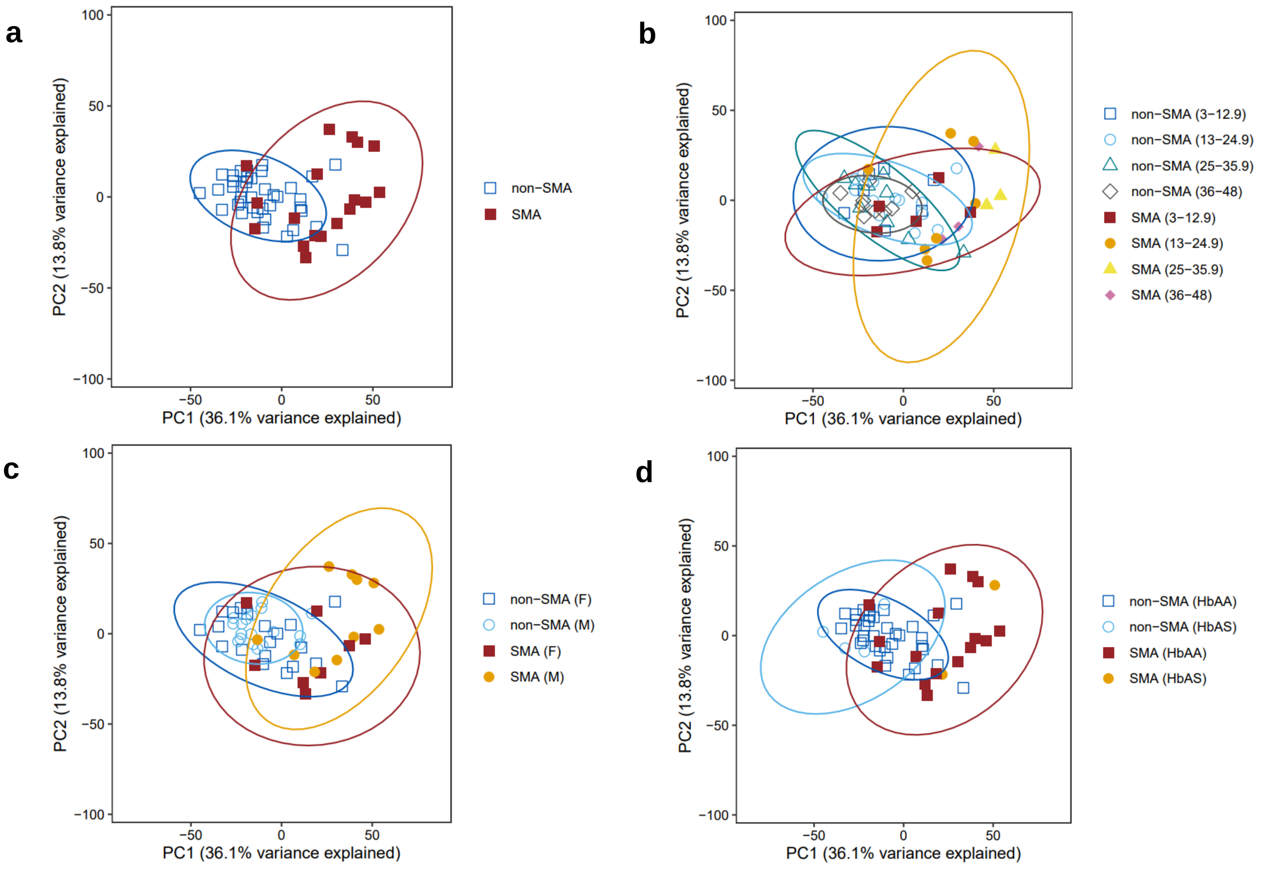

Fig. S3. Principal component analysis (PCA) for non-SMA and SMA. A PCA was created to investigate patterns of variance within children with non-SMA and SMA (57 observations (participants) of 1,682 variables (genes). The ellipses represent 95% confidence intervals for each cluster. (**a**) The PCA plot revealed that the first principal component (PCA1) accounted for 36.1% of the total variance, while the second principal component (PCA2) explained an additional 13.8% of the variance. PCA1 differentiated a portion of the non-SMA and SMA groups, suggesting that some underlying features are more pronounced in the SMA group. However, there was also some overlap between the two groups along this component. PCA2, despite accounting for a smaller proportion of the total variance, demonstrated a tighter clustering along the non-SMA group, suggesting higher variability in children with SMA. (**b**) Stratification of the non-SMA and SMA groups according to age revealed the tightest clustering among different ages within the non-SMA group, and greater dispersion in children with SMA, indicating more homogeneity within the age distribution of the non-SMA group. (**c**) Additional stratification between the two groups according to sex indicated the tightest clustering for males with non-SMA.

Greater dispersion was observed for females with SMA and was highest for males with SMA. (**d**) Stratification by sickle cell trait revealed the highest homogeneity for the non-SMA group with HbAA, followed by non-SMA with HbAS, and the highest dispersal within the SMA group for HbAA. There were not enough children with SMA carrying the HbAS trait to generate a cluster.

Abbreviations: non-SMA - non-severe malarial anemia (Hb≥6.0 g/dL), SMA - severe malarial anemia (Hb<6.0 g/dL), F - female, M - male, HbAA - normal hemoglobin carriers, and HbAS - sickle cell trait carriers.

Table S2. Top 10% of the up- and down-regulated blood-specific gene expression signatures.

	Position	Total Genes	Up (%)	Down (%)	Regulation (%)	Function	Differentially Expressed Genes in Dataset	DEG
Down-regulation	A33.6	18	0	76.923	-76.923	Inflammation	CD46, FCGR3B, HIF1A, LRRK2, MEFV, PHF20L1, RAB33B, SNAP23, SNX10, TLR1	10
	A28.1	21	0	76.190	-76.190	Interferon	DDX58, FBXO6, GBP1, GBP4, GBP5, IFI35, IFIH1, IFIT2, IFIT5, IRF7, LAP3, PARP14, SAMD9L, STAT1, TRIM22, ZBP1	16
	A28.3	31	0	75	-75.000	Interferon	ADAR, CASP1, CHMP5, DTX3L, GBP2, IFI16, IFITM1, IRF9, NOD2, PARP9, PSMB9, SP100, SP110, STAT2, TAP1, TDRD7, TMEM140, TRAFD1 , RIM21, UBE2L6, ZC3HAV1	21
	A28.2	15	0	54.545	-54.545	Interferon	ANKRD22, EIF2AK2, FCGR1B, PARP10, SP140, UBE2L6	6
	A35.10	65	0	51.923	-51.923	Neutrophil	ACSL4, DAM8, APBB1IP, BCL3, CDA, CPD, CYB5R4, EIF4E3, FCGR2A, FCGR3B, FPR1, HSPA1A, IFNAR1, LAT2, LITAF, LY96, NCF4, RALB, REPS2, RNF13, RNF149, RNF24, SDCBP, SOD2, STX11, TRIB1, VNN2	27
	A35.2	39	3.333	53.333	-50.000	Cytokines/ chemokines	BTNL8, CASP4, CASP5, CSF2RB, FFAR2, FPR2, GK, KCNJ15, KCNJ2, LIMK2, LRG1, MCTP2, NAMPT, NCF4, RNF149, SLPI, STEAP4	17
	A35.18	14	0	46.154	-46.154	TBD	BAZ1A, CD53, CKLF, GLRX, RHOT1, RNASEL	6
	A28.4	21	5.555	50.000	-44.444	Interferon	APOL2, COP1, DYNLT1, H2AFJ, ISG20, PSMB9, SP100, TNFSF13B, TRIM38, TRIM56	10
	A35.4	33	0	41.935	-41.935	Inflammation	FPR2, HIST1H2AC, HSD17B11, IL1B, LAT2, NCF2, NDUFB3, NT5C2, SOD2, TNFSF10, VNN2, WARS, WSB1	13
	A25.5	24	0	40.000	-40.000	Protein Phosphorylation	CLK1, HERC3, KIF5B, MORC3, NAT1, NLRC5, PDE4B, SPTLC1	8
	A35.1	20	0	38.462	-38.462	Cytokines/	LIMK2, MAPK14, OSM, S100P, ST3GAL4	5

	Position	Total Genes	Up (%)	Down (%)	Regulation (%)	Function	Differentially Expressed Genes in Dataset	DEG
						chemokines		
	A35.7	55	2.173	39.130	-36.957	Inflammation	ACSL1, ADM, AIM2, ANXA3, CEACAM1, CLEC4D, FCGR1A, FCGR1B, HPSE, IL1RN, KCNJ15, LIMK2, LMNB1, PGS1, PROK2, SIPA1L2, SLC26A8, TLR5, TNFAIP6	19
	A31.5	17	0	35.714	-35.714	TBD	HSPA6, NDEL1, REPS2, TNFAIP2, ZDHHC18	5
	A33.11	33	3.448	37.931	-34.483	Neutrophil	ARPC5, CAP1, FGD3, GMFG, HLA-E, IRF1, KPNB1, LCP1, MCL1, NFKBIA, PRR13, PTP4A2	12
	A35.5	16	0	33.333	-33.333	Inflammation	BMX, MAPK14, RAB20, SOCS3	4
	A17.3	15	0	33.333	-33.333	Gene Transcription	CACYBP, GPR27, SEMA6B, TBC1D20	4
Up-regulation	A3.9	31	31.578	5.263	26.316	TBD	ITPR1, NFATC3, NRBP2, SNX29, STX2, TSC22D2, TTC13	7
	A6.25	19	26.667	0.000	26.667	TBD	ATP2B1, BCOR, GFOD1, NFE2L1	4
	A37.8	17	26.667	0.000	26.667	Erythroid	HBBP1, RHD, TBCEL, YPEL4	4
	A36.6	14	27.272	0.000	27.273	Erythroid	GPX4, ST3GAL1, TM7SF2	3
	A8.7	42	28.571	0.000	28.571	TBD	AHDC1, MGAT4B, PCK2, POMZP3, RFNG, TAF6, TBC1D17, TTC7A	8
	A7.2	25	28.571	0.000	28.571	Monocyte	GPBAR1, PDK4, SH3TC1, TSPAN4, ZFHX3, ZNF385A	6
	A31.4	33	32.142	3.571	28.571	Platelet/ prostaglandin	CA2, GAS2L1, HIST2H2BE, MAP1A, NFIB, PEAR1, PNMA1, TAL1, TBXA2R, TGFB1I1	10
	A37.9	40	31.251	0.000	31.250	Erythroid	FURIN, GABARAPL2, GCLC, H1F0 MAF1, PINK1, PNPLA2, RNF14, STOM, TERF2IP	10
	A37.10	39	35.483	0.000	35.484	Erythroid	AMFR, GDE1, MED25, PSME4, RNF14, SLC2A1, TMEM183A, TMEM183B, WDR26, ZBTB44, ZNF653	11
	A36.4	17	36.363	0.000	36.364	Erythroid	AGBL5, CTSB, GABARAPL2, SH3GLB2	4
	A34.5	16	42.857	0.000	42.857	TBD	MGLL, MMRN1, SAMD14, SEC14L5, SELP, SYTL4	6

	Position	Total Genes	Up (%)	Down (%)	Regulation (%)	Function	Differentially Expressed Genes in Dataset	DEG
	A38.3	42	44.117	0.000	44.118	Erythroid	AP2S1, CARHSP1, CCNI, CLCN3, CTSB, CYB5R3, EIF4EBP2, ERAL1, GNAS, ISCU, MAP2K2, PQLC1, RFNG, TRIP12, UBE2M	15
	A31.8	17	46.153	0.000	46.154	TBD	FAM89B, LRRC8A, PSMD2, RAB1B, RAB5B, TBC1D10B	6
	A38.4	16	66.666	6.667	60.000	Erythroid	ARF1, ARL6IP1, ATP6V1D, EPN1, FDFT1, GHITM, HSPB1, MAP1LC3B, TAX1BP1, TPRG1L, YIPF3	11
	A38.2	13	70	0.000	70.000	Neutrophil Activation	CEACAM6, CEACAM8, CTSG, DEFA3, DEFA4, LTF, MPO	7
	A37.11	26	72.727	0.000	72.727	Erythroid	AP2M1, AP2S1, ATG9A, B4GALT3, BCL2L13, CYB5R3, DAP, HK1, JUND, NTAN1, PA2G4, UBAC1, UBL7, UROD, WDR13, WIPI2	16

Data are presented for the top 10% of upregulated and downregulated blood-specific signatures identified by the BloodGen3Module. The aggregates and sub-aggregates represent the position. The number of Total Genes comprising each module is shown, along with the up-regulated genes (Up %), down-regulated genes (Down %), and Regulation (%, difference between the two sets). The Function designates the biological process associated with the genes in each module. The actual genes within the set are shown as Differentially Expressed Genes in Dataset, along with the number of DEGs in the dataset.

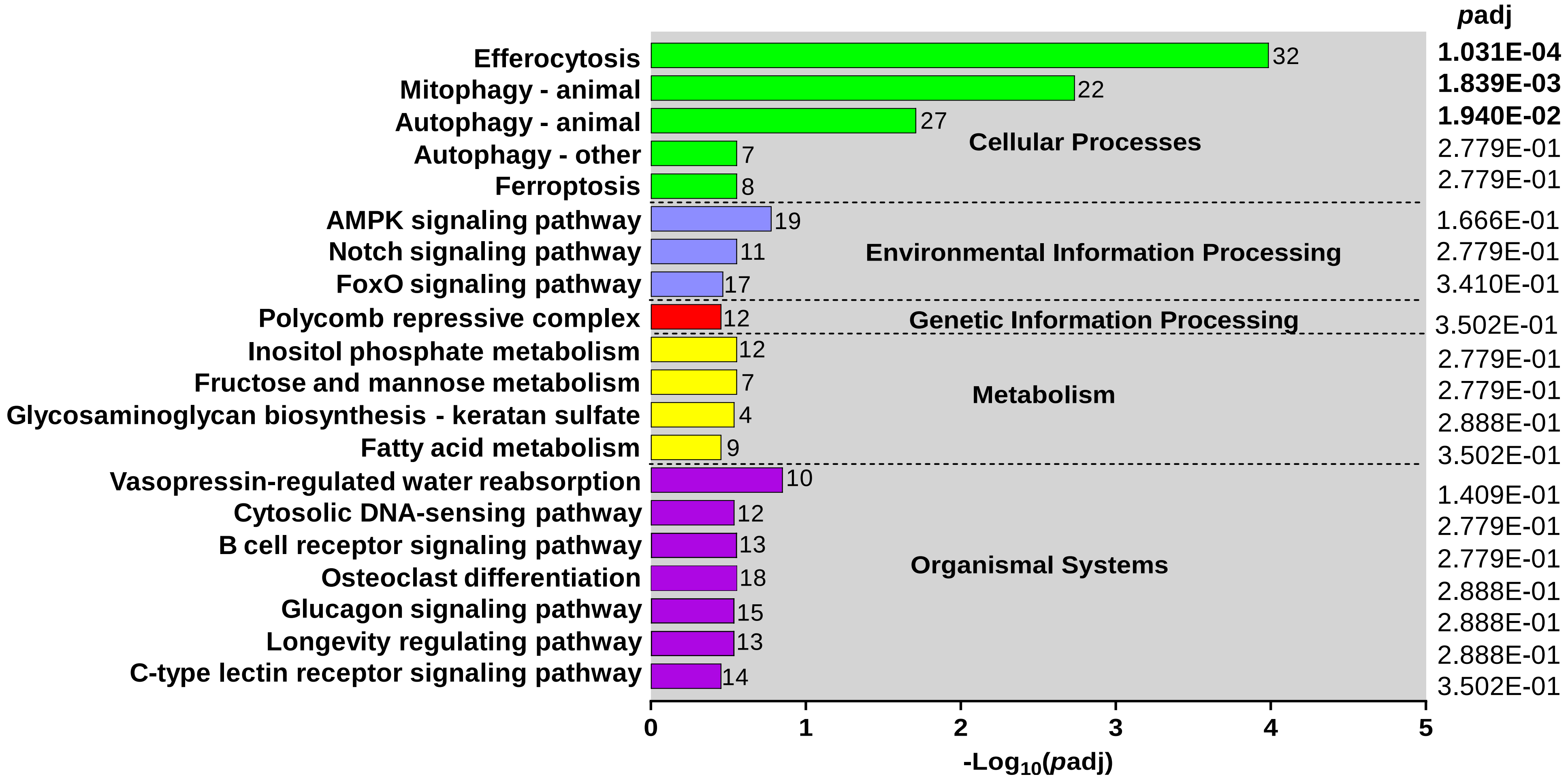

Fig. S4. Functional classification of KEGG pathways for the DEGs. The KEGG term category was Cellular Processes, Environmental Information Processing, Genetic Information Processing, Metabolism, and Organismal Systems. The left Y-axis shows the KEGG terms. The right Y-axis shows *p*-adjusted values for each KEGG term. The X-axis represents the negative log 10 of *p*-adjusted values [-Log_{10}(*p*adj)]. Bold indicates statistically

significant (*p*adj<0.050) pathways. Statistical significance was determined using one-sided overrepresentation analysis with multiple testing corrections using the Benjamini-Hochberg procedure.

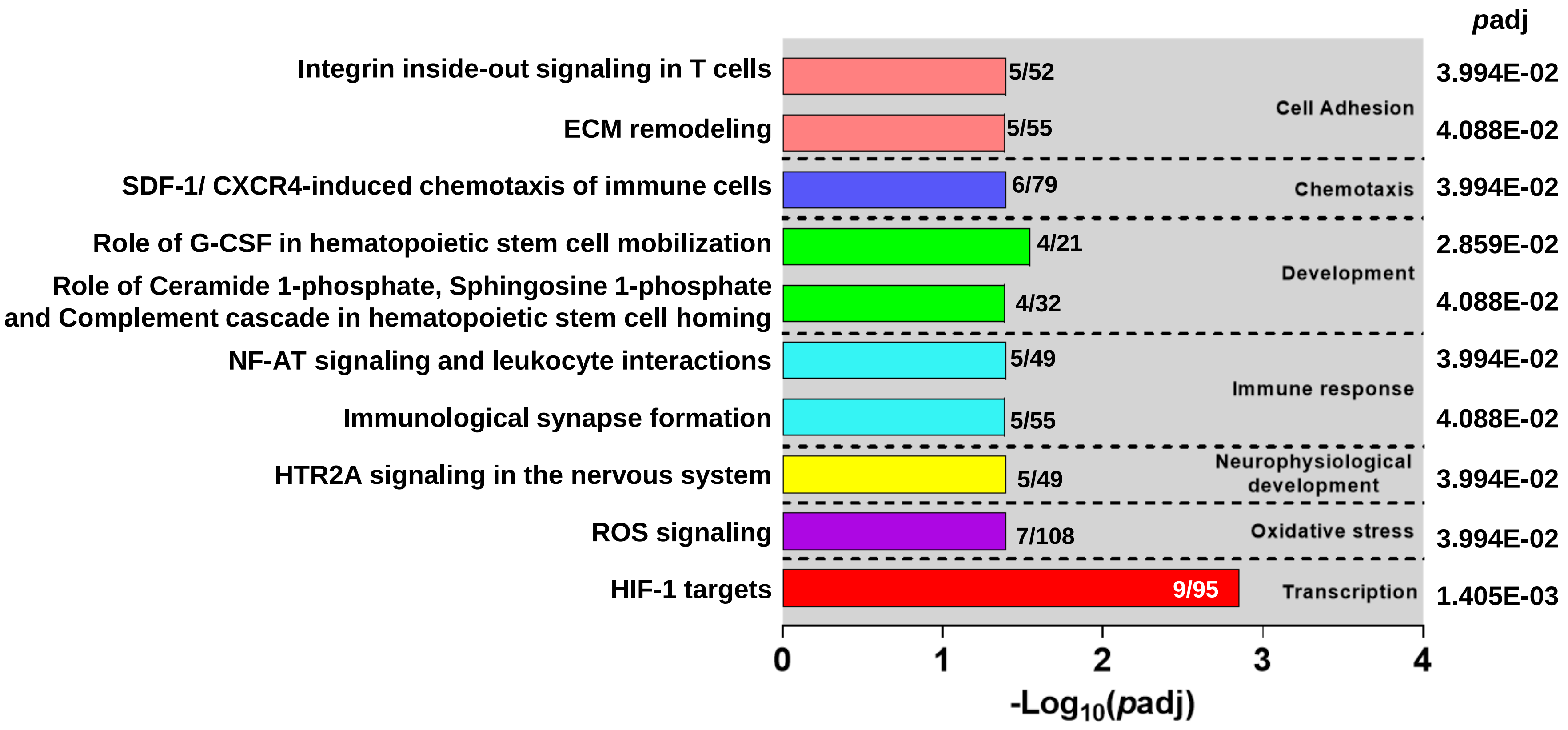

Fig. S5. Top-ranked Metacore™ canonical pathway maps for the Ugandan dataset. The top 10-ranked canonical pathway maps that emerged from an RNA-Seq analysis in a Ugandan cohort of children with SMA (n=17, case) and community children (household controls, n=12, control) according to *p*-adjusted values. The top 10 maps that emerged represent seven functional categories: (i) Cell Adhesion, (ii) Chemotaxis, (iii) Development, (iv) Immune Response, (v) Neurophysiological Development, (vi) Oxidative Stress, and (vii) Transcription. The left Y-axis indicates the specific biological pathways that were established by non-contradictory state-of-the-art knowledge of the major categories for human metabolism and

cell signaling. The right Y-axis shows *p*-adjusted values for each pathway map. The X-axis represents the $-\text{Log}_{10}(p\text{adj})$ value. Statistical test computed using a hypergeometric probability formula, and $p\text{adj}<0.050$. Bold indicates statistically significant ($p\text{adj}<0.050$) pathways.

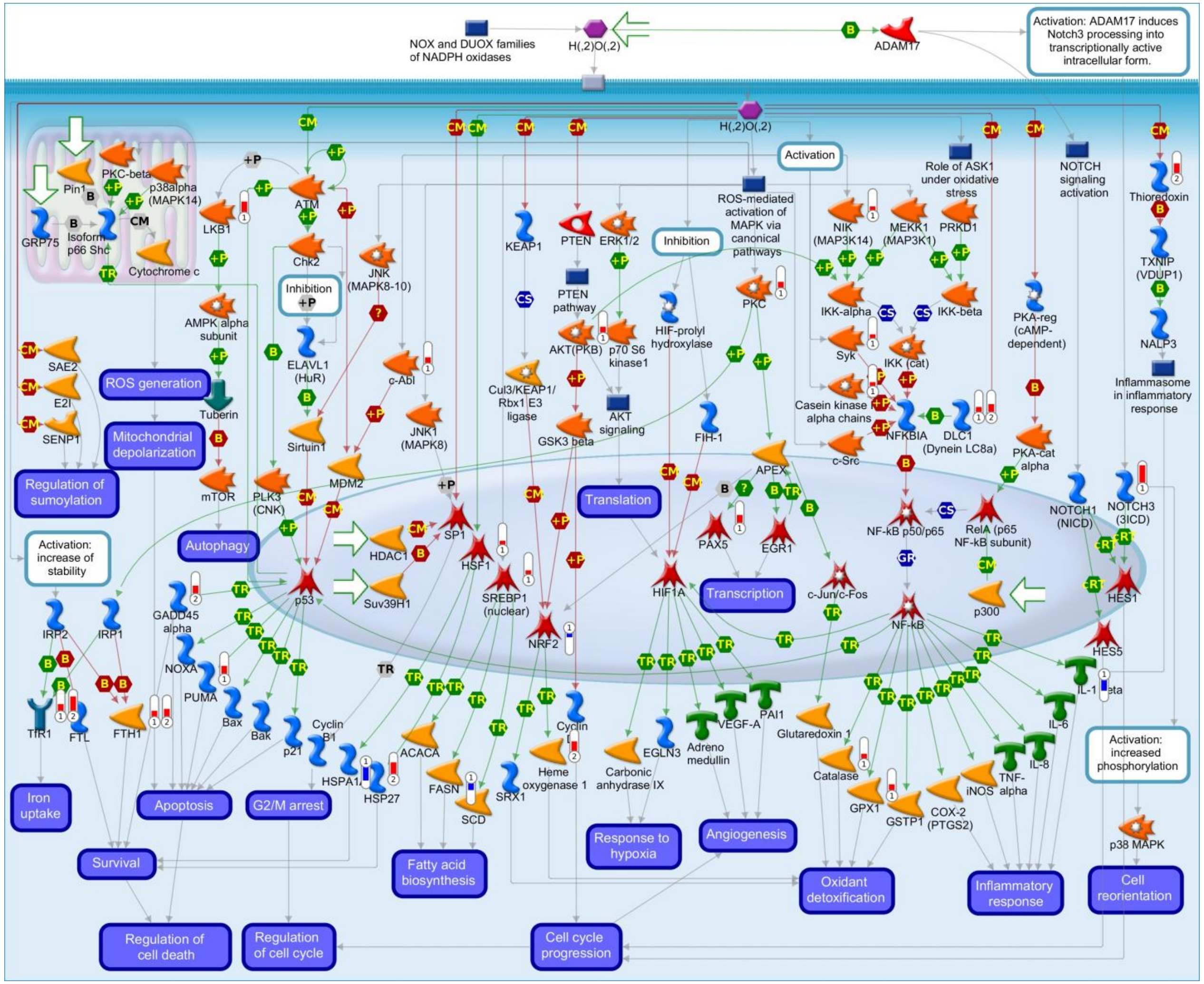
NOX and DUOX families of NADPH oxidases
H(,2)O(,2)
ADAM17
Activation: ADAM17 induces Notch3 processing into transcriptionally active intracellular form.
Pin1
PKC-beta
p38alpha (MAPK14)
GRP75
Isoform p66 Shc
Cytochrome c
LKB1
ATM
Chk2
Inhibition
JNK (MAPK8-10)
KEAP1
PTEN
ERK1/2
ROS-mediated activation of MAPK via canonical pathways
Activation
Role of ASK1 under oxidative stress
NOTCH signaling activation
Thioredoxin
TXNIP (VDUP1)
NALP3
Inflammasome in inflammatory response
NIK (MAP3K14)
MEKK1 (MAP3K1)
PRKD1
IKK-alpha
IKK-beta
Syk
IKK (cat)
PKA-reg (cAMP-dependent)
PTEN pathway
AKT(PKB)
p70 S6 kinase1
HIF-prolyl hydroxylase
PKC
AMPK alpha subunit
ELAVL1 (HuR)
c-Abl
SAE2
E2I
SENP1
ROS generation
Tuberin
Mitochondrial depolarization
Regulation of sumoylation
mTOR
PLK3 (CNK)
Sirtuin1
MDM2
JNK1 (MAPK8)
Cul3/KEAP1/ Rbx1 E3 ligase
GSK3 beta
AKT signaling
FIH-1
Casein kinase alpha chains
NFKBIA
DLC1 (Dynein LC8a)
PKA-cat alpha
c-Src
APEX
Translation
Autophagy
HDAC1
SP1
HSF1
PAX5
EGR1
NF-kB p50/p65
RelA (p65 NF-kB subunit)
NOTCH1 (NICD)
NOTCH3 (3ICD)
Activation: increase of stability
p53
Suv39H1
SREBP1 (nuclear)
HIF1A
Transcription
c-Jun/c-Fos
NF-kB
p300
HES1
HES5
IRP2
IRP1
GADD45 alpha
NOXA
PUMA
Bax
Bak
p21
Cyclin B1
HSPA1A
HSP27
NRF2
ACACA
FASN
SCD
SRX1
Cyclin D1
Heme oxygenase 1
Carbonic anhydrase IX
EGLN3
Adreno medullin
VEGF-A
PAI1
Glutaredoxin 1
Catalase
GPX1
GSTP1
COX-2 (PTGS2)
iNOS
TNF-alpha
IL-8
IL-6
IL-1 beta
Activation: increased phosphorylation
p38 MAPK
TfR1
FTL
FTH1
Iron uptake
Apoptosis
G2/M arrest
Survival
Fatty acid biosynthesis
Response to hypoxia
Angiogenesis
Oxidant detoxification
Inflammatory response
Cell reorientation
Regulation of cell death
Regulation of cell cycle
Cell cycle progression

Fig. S6. Common top-ranked Metacore™ canonical pathway map for the Kenyan and Ugandan datasets: ROS signaling. The second common top-ranked pathway map that emerged from RNA-Seq analysis in both the Kenyan cohort with SMA (n=18, case) and non-SMA (n=39, control) and a Ugandan cohort of children with SMA (n=17, case) and community children (household controls, n=12, control) was Reactive Oxygen Species (ROS) signaling involved in oxidative stress. The red thermometers indicate annotated genes that were up-regulated in children with SMA (1=Kenyan children and 2=Ugandan children), while the blue thermometers indicate down-regulated genes (1=Kenyan children and 2=Ugandan children). The details of symbols used in these figures are available at: https://portal.genego.com/legends/MetaCoreQuickReferenceGuide.pdf.

Table S3: Demographic and clinical characteristics of the study participants in the Qiagen Targeted RNA-Seq.

Characteristics	Total	non-SMA (Hb≥6.0 g/dL)	SMA (Hb<6.0 g/dL)	*p*-value
No. of participants, n	43	23	20	
Sex, n (%)				
Male	22 (51.2)	15 (65.2)	7 (65.0)	0.069[a]
Female	21 (48.8)	8 (34.8)	13 (35.0)	
Age, months	21.9 (22.9)	24.6 (21.2)	17.0 (16.3)	0.144[b]
0 - 12.9	11 (25.6)	5 (21.7)	6 (30.0)	
13 - 24.9	16 (37.2)	7 (30.5)	9 (45.0)	0.643[a]
25 - 35.9	7 (16.3)	5 (21.7)	2 (10.0)	
36 - 48.9	8 (18.6)	5 (21.7)	3 (15.0)	
>49	1 (2.3)	1 (4.4)	0 (0.0)	
Glucose, mmol/L	5.5 (3.2)	5.2 (3.5)	5.8 (3.2)	0.157[b]
Admission temperature, °C	37.8 (2.2)	37.8 (2.3)	37.7 (2.1)	0.893[b]
**Hematological Parameters**				
Hemoglobin, g/dL	9.7 (5.6)	10.3 (1.2)	4.6 (1.6)	NA
Hematocrit, %	29.8 (18.9)	33.9 (4.0)	15.1 (4.5)	**2.134E-08[b]**
Red blood cells, × 10^6/µL	4.2 (2.5)	4.7 (0.8)	2.2 (0.8)	**2.121E-08[b]**
Red cell distribution width, %	19.4 (4.1)	18.7 (2.7)	20.6 (5.5)	**0.006[b]**
Mean corpuscular volume, fL	70.9 (9.1)	70.2 (11.1)	72.0 (9.3)	0.679[b]
Mean corpuscular hemoglobin, pg	21.9 (3.3)	21.8 (4.5)	22.3 (3.5)	0.733[b]
Mean corpuscular hemoglobin concentration, g/dL	31.3 (2.1)	31.8 (2.2)	31.2 (2.6)	0.247 [b]
Platelets, ×10^3/µL	118.0 (159.0)	104.0 (156.0)	127.0 (164.2)	0.836[b]
Platelet distribution width, %	16.9 (1.8)	16.2 (1.3)	17.4 (1.2)	**0.017[b]**
Mean platelet volume, fL	8.2 (2.3)	7.8 (2.5)	8.5 (1.9)	0.733[b]
White blood cells, ×10^3/µL	12.0 (6.7)	11.7 (5.3)	15.1 (11.8)	0.134[b]
Lymphocytes, ×10^3/µL	4.1 (1.5)	4.0 (2.2)	5.0 (6.4)	0.036[b]
Monocytes, ×10^3/µL	0.9 (1.1)	0.9 (0.8)	1.0 (2.7)	0.457[b]
Neutrophils, ×10^3/µL	5.5 (3.2)	6.8 (0.0)	3.9 (0.0)	0.127[b]
Granulocytes, ×10^3/µL	7.3 (6.7)	6.9 (4.7)	9.9 (7.8)	0.131[b]
**Parasitological Indices**				
Parasite density, MPS/µL	46,398 (80,656)	44,723 (77,506)	48,982 (117,648)	0.450[b]
Low (1 – 5,000)	5 (11.6)	3 (13.0)	2 (10.0)	
Moderate (5,001 – 50,000)	18 (41.8)	10 (43.5)	8 (40.0)	0.826[a]
High (50,001 – 100,000)	10 (23.3)	6 (26.1)	4 (20.0)	
Hyper (>100,001)	10 (23.3)	4 (17.4)	6 (30.0)	
Geomean parasitemia, /µL	37,053	31,481	44,691	0.137[c]
**Clinical Complications**				
Respiratory distress	9 (20.9)	3 (13.0)	6 (30.0)	0.263[a]
Hypoxia, SpO_2 <90%	1 (2.3)	0 (0.06)	1 (5.0)	-
Convulsions	15 (34.9)	11 (47.8)	4 (20.0)	0.107[a]
Hypoglycemia (blood glucose levels <2.2 mM)	2 (4.8)	1 (4.3)	1 (5.3)	1.000[a]
Jaundice	3 (7.0)	0 (0.0)	3 (15.0)	0.092[a]
Thrombocytopenia (platelet count <150 × 10^3/mm^3	28 (65.1)	14 (60.9)	14 (70.0)	0.749[a]
**Genetic Variants**				
Sickle cell trait, n (%)				
Hb AA	40 (93.0)	20 (87.0)	20 (100.0)	0.236[a]
Hb AS	3 (7.0)	3 (13.0)	0 (0.0)	

Data are presented as number (percentages; %), median (interquartile range; IQR) or mean (standard error of mean; SEM) unless stated otherwise. Children (n=43) presenting with malaria were recruited at SCRH. Children were categorized into either non-severe malaria anemia (non-SMA; Hb≥6.0 g/dL, n=23) or severe malarial anemia (SMA; Hb<6.0 g/dL, n=20) based on hemoglobin (Hb) levels. Children presenting with sickle cell disease were excluded from the analysis. Sex (p=0.069), overall age (p=0.144), and distribution within age categories (p=0.643) were comparable within the groups. Further, there was no significant difference in glucose levels (p=0.157) and auxiliary temperature (p=0.893) within the SMA and non-SMA groups. The hematocrit levels (p=2.134E-08) and red blood cells (p=2.121E-08) were lower in children with SMA. In contrast, red cell distribution width (p=0.006) and platelet distribution width (p=0.017) were elevated in children with SMA. Other hematological parameters had no significant differences between the groups. Additionally, there were no notable distinctions observed in parasitological indices, clinical complications, and sickle cell trait status. [a]Fisher's exact test [presented as number (%)] with exact p-values for homogeneity was performed. [b]Two-sided Mann-Whitney-U tests [presented as median (IQR)] were used to compare the non-SMA and SMA groups, [c]Group means were compared by two-sided, two-sample t-test [presented as mean (SEM)], with equal variance. All p-values shown in bold remained below the significance level after multiple test corrections using the Bonferroni-Holm method (familywise error rate, significance level 0.050). Abbreviations: MPS - malaria parasites.

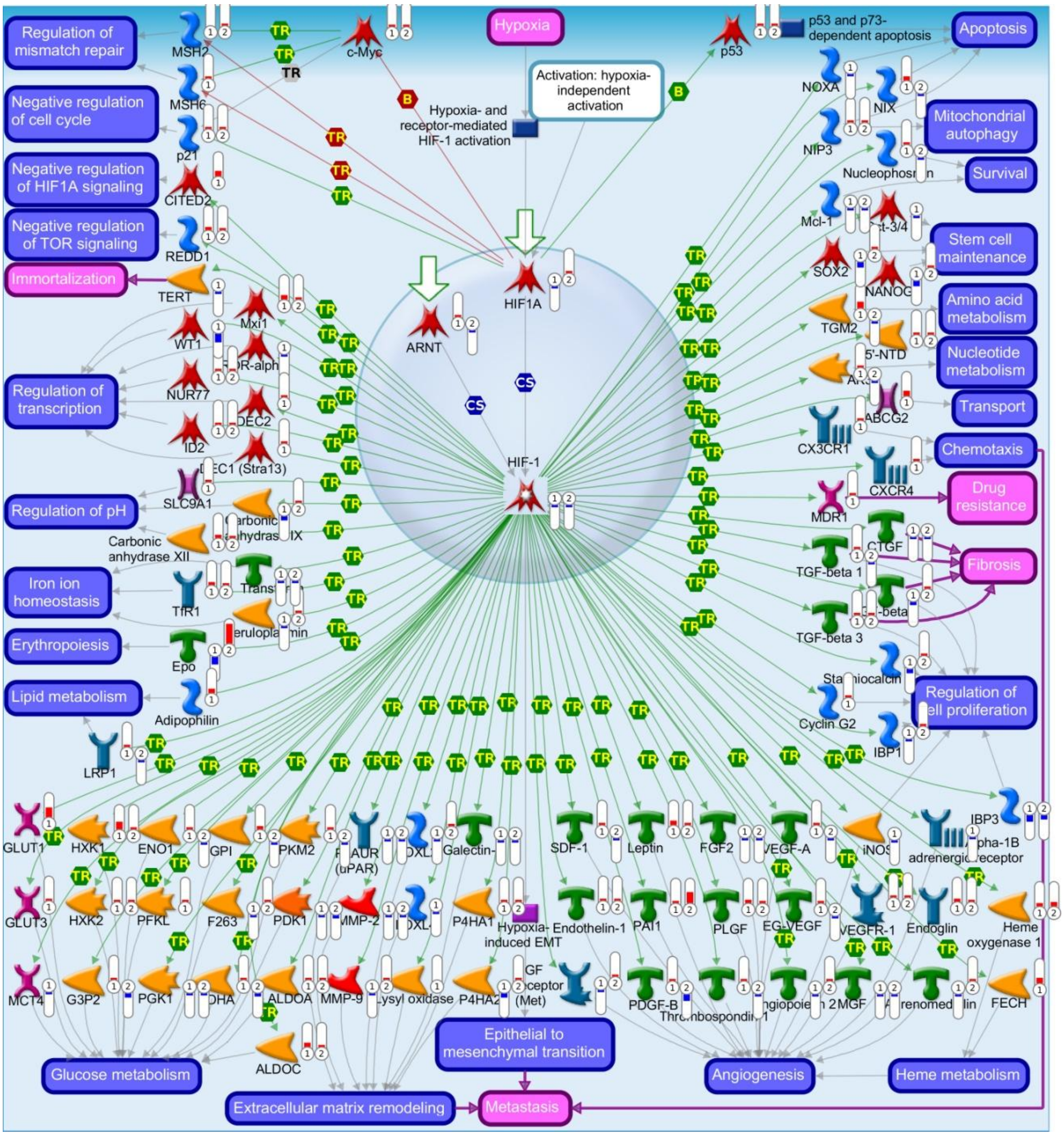

Fig. S7. Comparative analysis of transcript and protein abundance using Metacore™ canonical pathway maps. MetaCore™ was utilized to map genes with their corresponding protein products in 35 children (non-SMA, n=19 and SMA, n=16) with both RNA and protein measurements available. Pathway maps were generated without imposing thresholds to facilitate a comprehensive examination of all transcript/protein relationships. The most aligned (top-ranked) highly significant pathway for both the transcriptome and protein measurements was HIF-1 targets: 95/95 nodes for transcripts and 70/95 nodes for proteins, showing strong biological overlap for changes in the transcriptome mapping to protein abundance. The details of symbols used in these figures are available at: https://portal.genego.com/legends/MetaCoreQuickReferenceGuide.pdf.

Table S4. Proteome data standardization steps.

a. Sample normalization					
**Sample summary - Total number of samples and controls**					
	**Sample**	**QC**	**Buffer**	**Calibrator**	**Sum**
Total	40	6	5	5	56
Pass	34	6	5	5	50
Flag	6	0	0	0	6

**Hybridization Scale Factors (Accepted range,0.4-2.5)**			
**Assay Notes**	**Hyb. Scale Factor**	**Count**	**Notes**
Leak	3.88	1	Excluded from further analysis

**Adaptive Normalization by Maximum Likelihood (ANML) Fraction Used**			
**Dilution group**	**Pass**	**Flag**	**Total**
ANMLFractionUsed_20	40	0	40
ANMLFractionUsed_0_005	40	0	40
ANMLFractionUsed_0_5	40	0	40

**Median Normalization Scale Factors (Accepted range,0.4-2.5)**			
**Dilution group**	**Pass**	**Flag**	**Total**
NormScale_20	36	4	40
NormScale_0_005	38	2	40
NormScale_0_5	38	2	40

b. Calibration			
**Plate scale (Accepted range 0.4-2.5.)**			
**Plate**	**Acceptance Criteria**	**Plate Check**	**Value**
PLT14772	0.4 - 2.5	PASS	1.16

c. Quality Control (QC) Check			
**Calibrator percent in tails (Expected values outside the range of, 0.6 - 1.4)**			
**Plate**	**Guidance Criteria**	**Value**	
PLT14772	Less than 15%	2.9	

**SOMAmers in tails (Acceptance criteria, 0.8 - 1.2)**			
**SOMAmer**	**Acceptance Criteria**	**ColCheck**	**Count**
QC Ratio	0.8 - 1.2	PASS	7253
		FLAG	343
		TOTAL	7596

**QC Percent in tails (Accepted range, 0.8 - 1.2)**			
**Plate**	**Acceptance Criteria**	**Pass/Flag**	**Percent**
PLT14772	Less than 15%	PASS	4.5

**Calibrator Coefficient of variation (CV) - Distribution of Calibrator CVs on each plate**			
**Cal Precision (%)**	**10%**	**50%**	**90%**
PLT14772	1.4	3.0	7.6

**QC CVs - Percentiles distribution of QC CVs for all plates**				
**QC Lot**	**Number of Samples**	**10%**	**50%**	**90%**
200170	6	1.7	3.2	7.5

d. Sample Appearance		
**Sample Notes**	**Count**	**Notes**
Clogged/Low Volume	1	Excluded from further analysis

Standardization steps for the SomaScan assay proteome data. (**a**). Sample normalization - Summary shows the total number of samples and controls, including the number that passed or were flagged by the quality criteria. From the samples analyzed, one sample had a hybridization scale factor of 3.88 (accepted range of 0.4-2.5), suggesting a leak. The adaptive normalization by maximum likehood (ANML) fractions used was greater than 30%, indicating a pass for each

sample analyzed. The median normalization scale factors showed that 4 samples were not within the accepted range of 0.4-2.5, with three samples being excluded from analysis due to elevated median normalization scale across dilution factors. (**b**). Calibration – plate scaling was measured from the median of the set of calibrator reference ratios per plate, resulting in a single scale factor that was applied across the plate. The plate scale factor was 1.16 (acceptance range 0.4-2.5), indicating a pass. (**c**). Quality control check – <15% of the samples in the plate showed a calibrator percent of tails scale factor of 2.9, a value outside the accepted range of 0.6-1.4, suggesting a >75% pass. The SOMAmers in tails show the cumulative number of SOMAmer reagents in the QC control with a ratio on any plate. The flagged SOMAmer reagents (outside the accepted accuracy range of 0.8-1.2, n=343) were retained in the analysis since accuracy across all assays run is a robust quality metric but is not a requirement for identification of meaningful biological signals. The QC percent in tails (<15% of the samples in the plate) was 4.5, indicating a >75% pass. The distribution of the calibrator coefficient of variation (CVs) and the QC CVs shows 90% percentiles of 7.6 and 7.5, respectively. (**d**). Sample appearance – one sample noted as clogged/low volume had an elevated hybridization scale factor, and was excluded from further analysis.
